# Estimation of the shielding ability of a tungsten functional paper for diagnostic x‐rays and gamma rays

**DOI:** 10.1002/acm2.12122

**Published:** 2017-06-28

**Authors:** Hajime Monzen, Ikuo Kanno, Takahiro Fujimoto, Masahiro Hiraoka

**Affiliations:** ^1^ Graduate School of Medical Science Department of Medical Physics Kindai University Osakasayama Japan; ^2^ Graduate School of Medicine Department of Radiation Oncology and Image‐Applied Therapy Kyoto University Kyoto Japan; ^3^ Department of Nuclear Engineering Kyoto University Kyoto Japan; ^4^ Division of Clinical Radiology Service Kyoto University Hospital Kyoto Japan

**Keywords:** diagnostic imaging field, gamma rays, radiation shielding, tungsten functional paper (TFP), x‐rays

## Abstract

Tungsten functional paper (TFP) is a novel paper‐based radiation‐shielding material. We measured the shielding ability of TFP against x‐rays and gamma rays. The TFP was supplied in 0.3‐mm‐thick sheets that contained 80% tungsten powder and 20% cellulose (C_6_H_10_O_5_) by mass. In dose measurements for x‐rays (60, 80, 100, and 120 kVp), we measured doses after through 1, 2, 3, 5, 10, and 12 TFP sheets, as well as 0.3 and 0.5 mm of lead. In lead equivalence measurements, we measured doses after through 2 and 10 TFP sheets for x‐rays (100 and 150 kVp), and 0, 7, 10, 20, and 30 TFP sheets for gamma rays from cesium‐137 source (662 keV). And then, the lead equivalent thicknesses of TFP were determined by comparison with doses after through standard lead plates (purity >99.9%). Additionally, we evaluated uniformity of the transmitted dose by TFP with a computed radiography image plate for 50 kVp x‐rays. A single TFP sheet was found to have a shielding ability of 65%, 53%, 48%, and 46% for x‐rays (60, 80, 100, and 120 kVp), respectively. The lead equivalent thicknesses of two TFP sheets were 0.10 ± 0.02, 0.09 ± 0.02 mmPb, and of ten TFP sheets were 0.48 ± 0.02 and 0.51 ± 0.02 mmPb for 100 and 150 kVp x‐rays, respectively. The lead equivalent thicknesses of 7, 10, 20, and 30 sheets of TFP for gamma rays from cesium‐137 source were estimated as 0.28, 0.43, 0.91, and 1.50 mmPb with an error of ± 0.01 mm. One TFP sheet had nonuniformity, however, seven TFP sheets provided complete shielding for 50 kVp x‐rays. TFP has adequate radiation shielding ability for x‐rays and gamma rays within the energy range used in diagnostic imaging field.

## 1 INTRODUCTION

Lead aprons are widely used as radiation protection devices in medical care because of their excellent shielding ability against x‐rays and gamma rays. However, workers who regularly wear heavy lead aprons may experience back pain or disk disease.^1^, ^2^ Other shortcomings of aprons made of lead or lead equivalent materials include inflexibility and toxicity.^3^, ^4^ To overcome these problems, some researchers have explored ways to provide effective x‐ray protection using alternative materials.^5^, ^6^, ^7^, ^8^ In an attempt to reduce the weight of protection materials, several vendors have developed composite lead‐equivalent materials using mixtures of different elements such as lead, tin, copper, bismuth‐antimony, and yttrium. These composite materials have good shielding capabilities against diagnostic x‐rays.^9^, ^10^ For example, bismuth‐antimony aprons have been widely used as lead‐free radiation protection materials in medical situations. The only disadvantages reported in humans were effects on the skin and eyes from acute exposure to antimony. The skin effects consist of a condition known as antimony spots, which are rashes formed by pustules around sweat and sebaceous glands, while effects on the eye include ocular conjunctivitis. Oral exposure to antimony has also resulted in gastrointestinal effects in humans.^11^, ^12^

The European Union directive “Restriction of the Use of Certain Hazardous Substances” for electrical and electronic equipment, prohibited the use of lead in electrical appliances after July 1, 2006.^13^, ^14^ There is therefore a strong demand for lead‐free product development in healthcare and industrial applications.

For the production of lead‐free radiation‐shielding materials, we have developed a paper‐based radiation‐shielding product containing tungsten: tungsten functional paper (TFP).^15^ In the previous study, we reported on the electron beam shielding ability of TFP, and TFP was shown to be a particularly promising material for protection from electron beam radiation.^15^

The purpose of this study was to estimate the x‐ray radiation shielding ability of TFP within the range of x‐ray tube voltages commonly used in diagnostic imaging (60–120 kVp). The lead equivalence of the TFP was also measured using well‐characterized x‐rays and gamma rays.

## 2 MATERIALS AND METHODS

### 2.A Characteristics of TFP

The TFP was prepared by Toppan Printing (Tokyo, Japan) and supplied in 0.3‐mm‐thick sheets that contained 80% tungsten powder by mass. The element ratios in the TFP (mol%) were H: 24.2%, C: 40.4%, O: 20.2%, and W: 15.2%.^15^ The physical properties of the TFP, which included dry mass, thickness, tensile strength, wet strength, tear strength, and air resistance, were in compliance with the Japanese Industrial Standards for paper manufacturing, as shown in Table 1.^16^, ^17^, ^18^, ^19^, ^20^, ^21^ The TFP had feature to pass through the 100 ml air within 67 s. The TFP was found to be a weak material in water: the tensile strength was 118N/15 mm in dry condition, however, the strength in wet condition was 5.9 N/15 mm.

**Table 1:** Physical properties of TFP. Measurement methods for each property are listed in the right‐most columns

Bone‐dry basis weight	(g m^−2^)	700	JIS‐P8124
Thickness	(mm)	0.3	JIS‐P8119
Tensile strength	(N/15 mm)	118	JIS‐P8113
Wet strength	(N/15 mm)	5.9	JIS‐P8135
Tear strength	(mN)	220	JIS‐P8116
Air strength	(s/100 mL)	67	JIS‐P8117

### 2.B Shielding rate measurements

**Figure 1:**
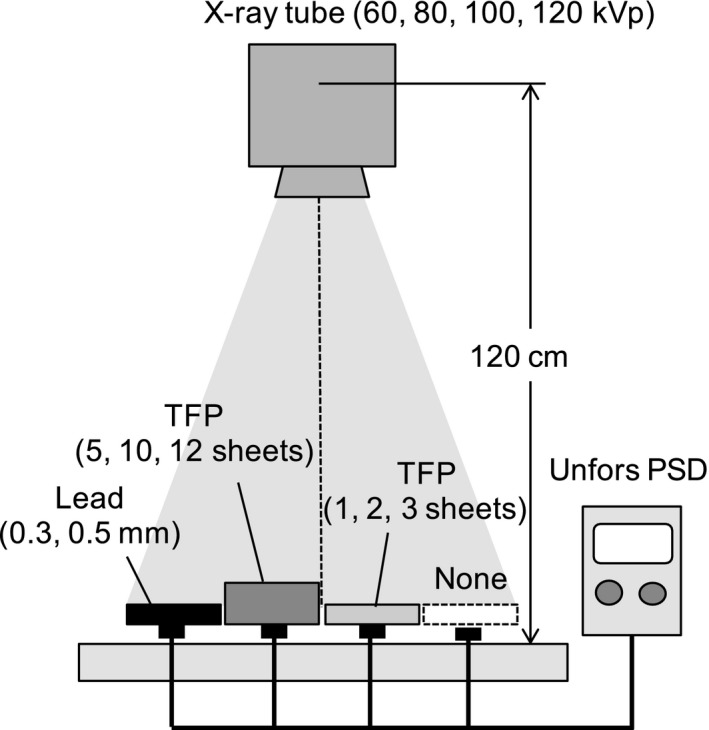
Schematic drawing of the experimental setup used for x‐ray dose measurement after passing through: air only, 1–12 sheets of TFP, and 0.3‐ and 0.5‐mm thicknesses of lead.

An x‐ray tube (UD‐150B‐40, Shimadzu, Kyoto, Japan) was used to generate x‐rays in the diagnostic range. An Unfors Patient Skin Dosimeter (PSD; Unfors Instruments AB, Billdal, Sweden) was used for dose measurements: this device was capable of measuring four skin doses by simultaneous use of four detectors. The experimental setup is shown in Fig. 1. The doses given by the diagnostic x‐rays were measured after passing through only air, and through 1, 2, 3, 5, 10, and 12 sheets of TFP, as well as 0.3 and 0.5 mm of lead. X‐ray tube voltages of 60, 80, 100, and 120 kVp were tested, with a tube current of 200 mA and an exposure time of 40 ms.

### 2.C Lead equivalence measurements

**Figure 2:**
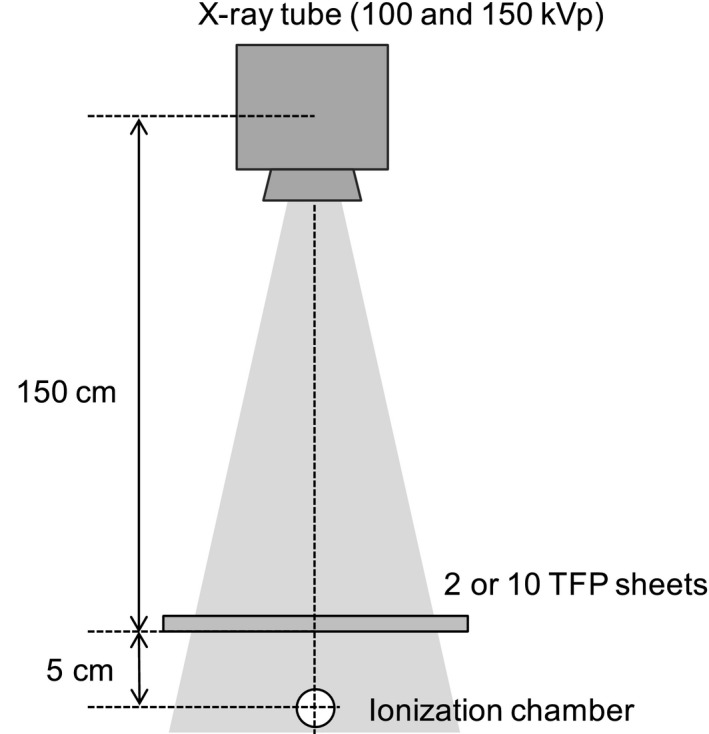
Schematic drawing of experimental setup used for lead equivalence measurements of TFP sheets using x‐rays.

The lead equivalences of the TFP for x‐ray and gamma ray shielding were measured according to the Japanese Industrial Standard JIS Z 4501.^22^ The highly characterized x‐ray beam and gamma ray sources of the Ionizing Radiation Standards Group at the Tokyo Metropolis Industrial Technology Research Institute were used for this purpose. For x‐ray shielding measurement, an MG‐452 x‐ray tube (Yxlon International Co., Ltd., Beijing, China) was operated at two tube voltages, 100 and 150 kVp. Either two or ten sheets of TFP were tested, as shown in Fig. 2. A Ramtec‐1000D A‐4 probe (Toyo Medic Co., Ltd. Tokyo, Japan) ionization chamber radiation dosimeter was used for the measurement of the lead equivalence of the TFP sheets.

**Figure 3:**
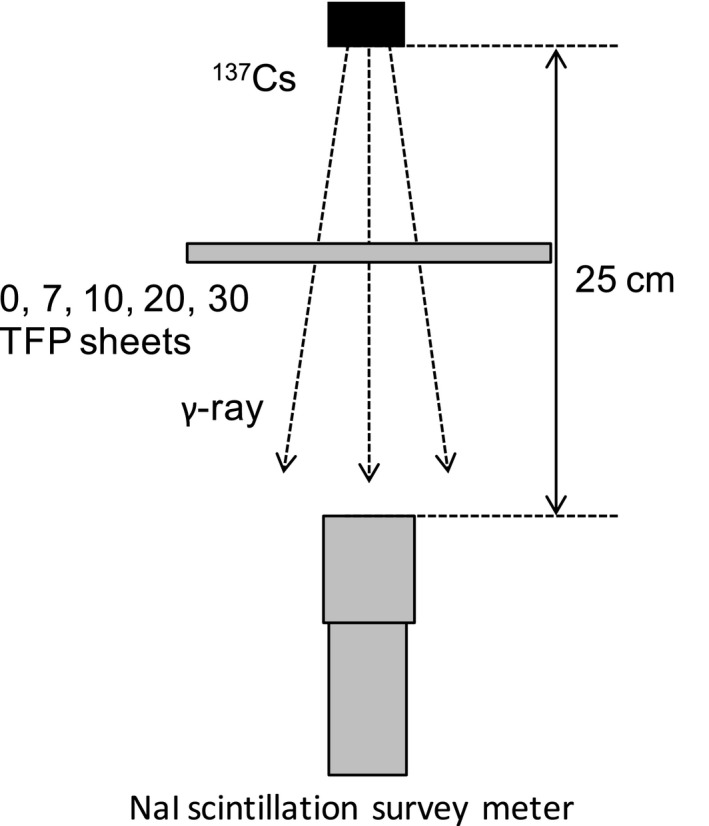
Schematic drawing of experimental setup for lead equivalence measurements of TFP sheets using gamma rays.

Gamma ray shielding was measured using a 100 MBq (Dec. 16, 2009) cesium‐137 source as the gamma ray emitter, and either 0, 7, 10, 20, and 30 sheets of TFP. The gamma rays passing through the TFP sheets were measured using a TCS‐171B NaI scintillation survey meter (Aloka Co., Ltd., Tokyo, Japan). The experimental set up is shown in Fig. 3.

Standard lead plates with thicknesses of 0.05, 0.11, 0.20, 0.31, and 0.50 mm, were used to measure attenuation rates through lead. The purity of the standard lead plates was not less than 99.9% and the thicknesses were chosen to have attenuation ratios from approximately half that of two TFP sheets to double that of 30 TFP sheets. An attenuation rate curve was prepared using the data from the standard lead plates and quadratic interpolation. The lead equivalent thicknesses of TFP for x‐rays and gamma rays were then obtained according to the interpolated measurement data.

### 2.D Evaluation of dose uniformity within a shielded region

**Figure 4:**
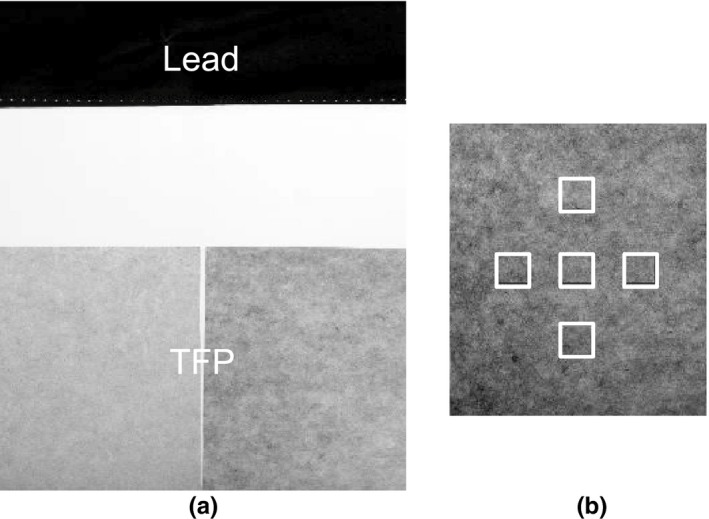
Schematic drawing of the arrangement of shielded regions on the CR plate used for uniformity measurements. (a) The positions of the lead and TFP sheets on the CR. (b) The regions‐of‐interest defined for measuring pixel values within the TFP‐shielded region.

The uniformity of the transmitted dose within a region shielded by the TFP was characterized using a computed radiography (CR) image plate (24 × 30 cm, FCR XG 5000 Plus, Fujifilm, Tokyo, Japan). From one to seven sheets of TFP, and a 0.25‐mm thickness of lead, were placed on the CR image plate as shown in Fig. 4(a). The section of the CR image plate between the lead and TFP sheets received direct unshielded x‐rays. Images were acquired using the tube voltage of 50 kVp, the current of 200 mA, and the exposure time of 10 ms. Five regions‐of‐interest (ROIs) were defined within the TFP‐shielded region, as shown in Fig. 4(b). The means and standard deviations of the pixel values were calculated using MATLAB (MathWorks, Natick, MA, USA). The mean value of pixels within an ROI was expected to be closely related to the number of x‐rays transmitted through the lead or TFP sheets. The standard deviation of the pixel values within an ROI represented the nonuniformity of the TFP sheets.

## 3 RESULTS

### 3.A Shielding rate and lead equivalence

**Figure 5:**
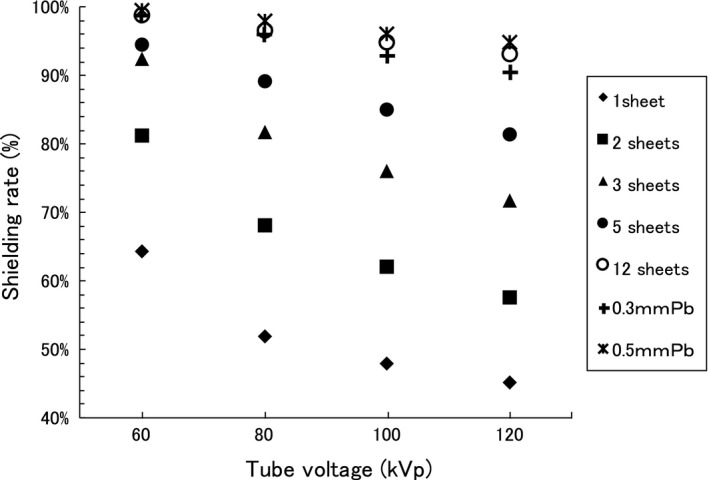
Shielding rate of 1–12 TFP sheets as a function of tube voltage. The shielding rates of 0.3‐ and 0.5‐mm thicknesses of lead are also shown. The statistical uncertainties of each measurement were within ±1.0%, which were defined the standard deviations of five times measurements.

A shielding rate was defined by the ratio of the transmission radiation (mGy) with TFP to the one without TFP. Figure 5 shows that one sheet of TFP provided x‐ray shielding rates of 65%, 53%, 48%, and 46%, at tube voltages of 60, 80, 100, and 120 kVp, respectively. The shielding rates measured through 1–12 sheets of TFP were not proportional to the tube voltage, as the dependence of the mass attenuation coefficient on the photon energy does not show a linear relationship.

Table 2 lists the lead equivalence of the TFP for shielding against x‐rays and gamma rays.

**Table 2:** Lead equivalences (mmPb) for the numbers of TFP sheets used for shielding against 100 kVp and 150 kVp x‐rays, and ^137^Cs γ‐rays

Number of sheets	Basis weight (kg m^−2^)	X‐ray energy (kVp)		^137^Cs γ‐ray energy (keV)
		100	150	662
2	1.4	0.10^a^	0.09^a^	−
7	4.9	−	−	0.28^b^
10	7	0.48^a^	0.51^a^	0.43^b^
20	14	−	−	0.91^b^
30	21	−	−	1.50^b^

a with an uncertainty of ±0.02 mm.

b with an uncertainty of ±0.01 mm.

### 3.B Evaluation of dose uniformity within the shielded region

**Figure 6:**
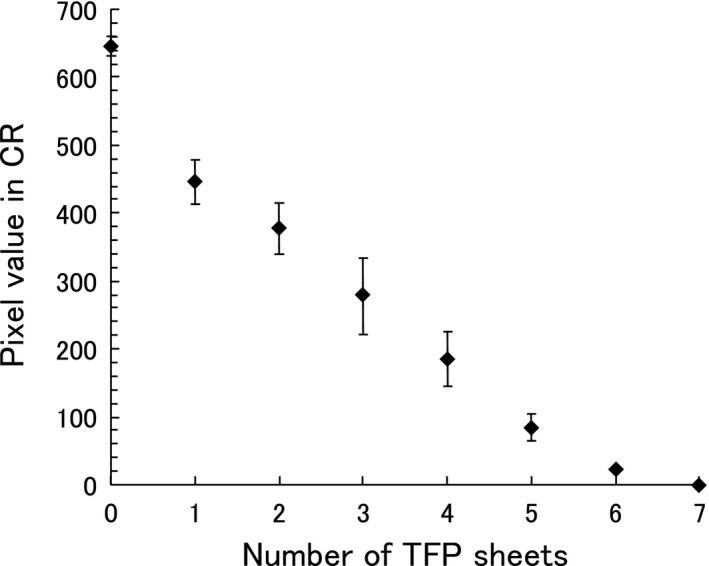
Pixel values of CR as a function of the number of TFP sheets (irradiated by 50 kVp x‐rays).

The mean pixel values and standard deviations measured by the CR image plate are shown as a function of the number of TFP sheets in Fig. 6. These were obtained using x‐rays generated with a tube voltage of 50 kVp. The means and standard deviations of pixel values in the nonshielded and 0.25‐mm‐thick lead‐shielded regions were 645.9 ± 7.1 and 3.0 ± 5.6, respectively. The standard deviation of pixel values in the TFP‐shielded region was larger than that in the non‐shielded region (i.e., zero TFP sheets). This was due to the nonuniformity of the TFP sheets. Seven sheets of TFP provided complete x‐ray shielding at 50 kVp.

## 4 DISCUSSION

We characterized the radiation shielding performance of TFP and found that 12 sheets of TFP had approximately the same shielding ability as 0.3–0.5 mm of Pb for x‐rays typical of those used in medical diagnosis (Fig. 5).

**Figure 7:**
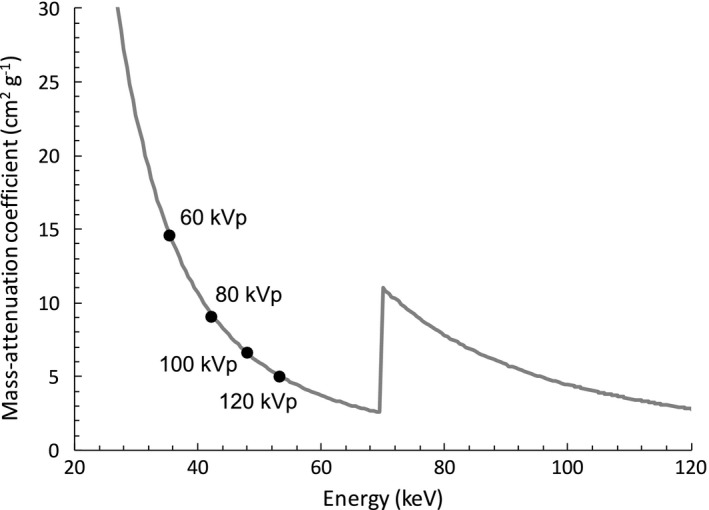
Mass attenuation coefficient of tungsten (solid line) and the averaged energies of x‐ray tube voltages 60, 80, 100, and 120 kVp.

The shielding rate for 60 kVp x‐rays are greater than those for 80, 100 120 kVp x‐rays, as shown in Fig. 5. This behavior is significant for less numbers of TFP. The shielding ability of white x‐rays is equal to the one of x‐rays with averaged energy over the x‐ray spectrum. The averaged energies for the x‐rays with tube voltages 60, 80, 100 and 120 kVp are, for example, 35.6, 42.4, 48.2 and 53.4 keV, respectively, with the conditions of tungsten target angle 22° and 2.5 mm thick Al filter. The mass attenuation coefficient of tungsten^5^ is shown in Fig. 7, as well as the averaged x‐ray energies described above. Because the curve of mass attenuation coefficient at lower x‐ray energy is steep, the shielding rate for 60 kVp x‐rays has much higher value than the ones for x‐rays with higher tube voltages. As the number of TFP sheets increases, x‐rays with low energy are absorbed much, and the averaged energy for 60 kVp x‐rays will increase. As a result, the shielding rate for 60 kVp with 5 and 12 TFP sheets don't show much higher value from the ones for x‐rays with higher tube voltages.

The TFP has a nonuniform tungsten particle distribution, that is, there are regions containing paper fibers, and regions containing the tungsten particles. The number of tungsten particles per unit area becomes more uniform when TFP sheets are laminated, which also increases the shielding rate. Figure 6 demonstrates that six or seven sheets of TFP are sufficient to create acceptable levels of uniformity.

## 5 CONCLUSIONS

We describe a novel lead‐free radiation‐shielding material, TFP, and characterize its radiation shielding ability for x‐rays within the energy range used in diagnostic imaging (60–120 kVp). The lead equivalence of TFP was estimated using well‐characterized x‐rays and gamma rays. TFP has potential applications as a radiation‐shielding material and has several benefits, including the fact that it is easy to process and can be folded, cut using scissors, and affixed to other materials. TFP is lead‐free, which allows to be used in applications where skin contact with the shielding material may occur and also suitable for use both indoors and outdoors.

## ACKNOWLEDGMENTS

This work was supported by the JSPS KAKENHI grant numbers 25461877 and 16K09027. We thank Mr. Masaru Hayakawa and Mr. Yuji Miki for their valuable support.

## CONFLICT OF INTEREST

Hajime Monzen has a consultancy agreement with, and financial interest in, TOPPAN PRINTING CO., LTD, Tokyo.
